# Cell type‐specific DNA methylation analysis of the prefrontal cortex of patients with schizophrenia

**DOI:** 10.1111/pcn.13282

**Published:** 2021-07-22

**Authors:** Junko Ueda, Miki Bundo, Yutaka Nakachi, Kiyoto Kasai, Tadafumi Kato, Kazuya Iwamoto

**Affiliations:** ^1^ Department of Molecular Brain Science Graduate School of Medical Sciences, Kumamoto University Kumamoto Japan; ^2^ Laboratory for Molecular Dynamics of Mental Disorders, RIKEN Center for Brain Science Saitama Japan; ^3^ Department of Neuropsychiatry Graduate School of Medicine, The University of Tokyo Tokyo Japan; ^4^ The International Research Center for Neurointelligence (WPI‐IRCN) The University of Tokyo Institutes for Advanced Study (UTIAS) Tokyo Japan; ^5^ Department of Psychiatry and Behavioral Science Graduate School of Medicine, Juntendo University Tokyo Japan

Epigenetics reflects complex interactions between genes and the environment and plays a role in long‐lasting gene expression changes. Therefore, unraveling epigenetic landscape of brain cells from patients with psychiatric disorders will contribute to understanding the pathophysiology of these disorders.^1^ We performed promoter‐wide DNA methylation analysis of the prefrontal cortex of patients with schizophrenia (N = 35, Table S1). Brain cells were separated into neuronal and nonneuronal nuclei by NeuN‐based nuclei sorting.^2^ Methylated DNA was collected using the MBD2B/3L, which does not bind hydroxymethylcytosine and enables the analysis of densely methylated regions in the genome. DNA methylation profiles were obtained with promoter tiling arrays covering 25 500 human promoters. Differentially methylated regions (DMRs) were identified by using a deposited control dataset (N = 35, GSE137921), which had been obtained by the same experimental procedure. This study was approved by the ethics committees of the participating institutes (the Research Ethics Committee of Kumamoto University, the Research Ethics Committee of the Faculty of Medicine of The University of Tokyo, the Ethical Review Board of Juntendo University, and the Wako 1st Research Ethics Committee of RIKEN). This study was conformed to the provisions of the Declaration of Helsinki. The experimental procedures are described in Appendix S1.

We identified 91 DMRs and 69 DMR‐associated genes in nonneurons and 74 DMRs and 59 DMR‐associated genes in neurons (Tables S2 and S3). We found that 59.4% of nonneuronal and 69.5% of neuronal DMR‐associated genes were shared between the two cell types (Fig. 1a). The DMR with the most significant change in neurons was located in the *CHGA* promoter region and showed hypermethylation. This DMR was also identified and showed the most significant change in nonneurons. *CHGA* encodes chromogranin A, a neuroendocrine protein located in the vesicles of neurons. The level of chromogranin A was reported to be reduced in the cerebrospinal fluid and prefrontal cortex in schizophrenia.^3^, ^4^ The DMR with the second highest score in neurons was found in the *LINGO1* promoter region, and showed hypermethylation. This DMR was also identified and showed the fifth highest change in nonneurons. LINGO1 plays a role in myelination and neurite outgrowth, and the disturbance of LINGO1 signaling has been implicated in the pathophysiology of schizophrenia.^5^ Gene ontology analysis using both neuronal and nonneuronal DMR‐associated genes revealed the enrichment of potassium channel‐related terms (FDR corrected *P* < 0.05) (Fig. 1b). In addition, enrichment of the Ras/Rho signaling and glucocorticoid receptor signaling pathways was detected (nominal *P* < 0.05).

**Fig. 1:**
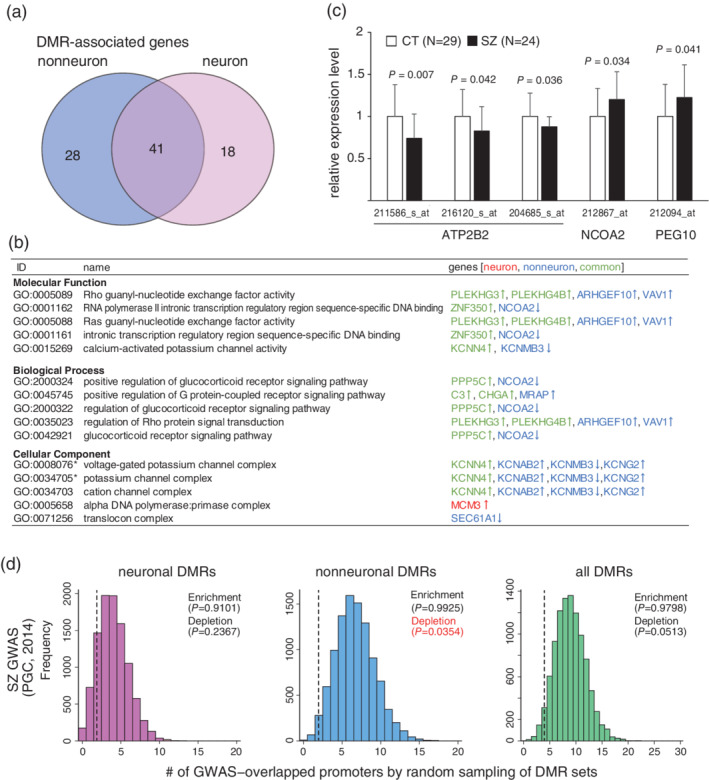
Differentially methylated regions (DMRs) in schizophrenia. (a) Venn diagram of DMR‐associated genes. (b) Gene ontology analysis of DMR‐associated genes. The top five terms in each category are shown. * indicates FDR‐corrected *P* < 0.05. Green, blue, and red indicate common, nonneuronal, and neuronal DMR‐associated genes, respectively. Arrows show the direction of change in schizophrenia. See Table S4 for details. c. Relative expression levels of DMR‐associated genes. Gene expression levels were measured in a previous microarray study (Appendix S1). Subjects with low brain pH were omitted from data analysis. Bars indicate standard deviations of the means. CT, control; SZ, schizophrenia. d. Enrichment and depletion between the DMRs and GWAS loci, assessed by random sampling. The frequency was based on 10,000 random samplings of DMR sets. See Fig. S1 for the results for other GWAS loci. *P*‐values in red indicate the significant depletion of DMRs.

We previously performed a microarray‐based gene expression analysis of the same brain region in the same subjects.^6^ By utilizing the dataset we assessed the gene expression status of DMR‐associated genes. We found that the expression levels of 25 probes, which covered 19 DMR‐associated genes, showed reliable gene expression values. Among them, five probes for three genes (*ATP2B2*, *NCOA2*, and *PEG10*) showed significantly altered expression (Welch's t‐test, nominal *P* < 0.05) (Fig. 1c). *ATP2B2* encodes plasma membrane calcium ATPase isoform 2 and *de novo* damaging variants in this gene were identified in autism.^7^ *NCOA2* encodes nuclear receptor coactivator 2 and has a histone acetyltransferase activity. *PEG10* encodes paternally imprinted gene 10 and contains two overlapping open reading frames, RF1 and RF2. *PEG10* was also identified as a differentially expressed gene in schizophrenia in a large‐scale transcriptomics study.^8^ The hypermethylated DMRs in schizophrenia are located in the 3'‐UTR of *PEG10* and may affect the regulation of isoform variations.

We then assessed whether the DMRs were enriched in genome‐wide association study (GWAS) loci of psychiatric disorders by promoter‐based random sampling analysis (Appendix S1). We previously found that neuronal DMRs in bipolar disorder (BD) were significantly enriched in the GWAS loci of BD, but nonneuronal DMRs were depleted from the GWAS loci of schizophrenia.^9^ In this analysis, we found no significant enrichment or depletion of the DMRs in the GWAS loci of any psychiatric disorders (*P* > 0.05). To increase sensitivity, we defined the DMRs with a relaxed threshold (*P* < 10^−5^), yielding 525 neuronal and 958 nonneuronal DMRs. Similar to the DMRs in BD, we found significant depletion of the nonneuronal DMRs from the schizophrenia GWAS loci^10^ (*P* = 0.0354), whereas no enrichment or depletion in the neuronal DMRs (Figs 1d and S1). These results suggest that the DMRs, especially the nonneuronal DMRs, and the GWAS loci of schizophrenia may have different spatiotemporal roles in the brain.

In summary, we identified DMRs of the prefrontal cortex of patients with schizophrenia. The DMRs reported in this study will be useful for understanding the pathophysiology of schizophrenia.

## Disclosure statement

Dr. Kasai reports grants from AMED, grants from JSPS KAKENHI, during the conduct of the study; grants from Lily, grants from MSD, grants and personal fees from Astellas, grants and personal fees from Takeda, grants and personal fees from Dainippon Sumitomo, grants from Novartis, grants from Tanabe‐Mitsubishi, grants from Eisai, grants and personal fees from Otsuka, grants from Shionogi, grants from Ono Pharma, personal fees from Fuji‐film‐Wako, personal fees from Yoshitomi, personal fees from Kyowa, personal fees from Janssen, personal fees from Meiji Seika Pharma, outside the submitted work. Dr. Kato reports grants and personal fees from the Japan Agency for Medical Research and Development, grants and personal fees from Ministry of Education, Culture, Sports, Science and Technology (MEXT)/Japan Society for the Promotion of Science (JSPS), during the conduct of the study; personal fees from Kyowa Hakko Kirin Co., Ltd., personal fees from Eli Lilly Japan K.K., grants and personal fees from Otsuka Pharmaceutical Co., Ltd., personal fees from GlaxoSmithKline K.K., personal fees from Taisho Pharma Co., Ltd., grants and personal fees from Dainippon Sumitomo Pharma Co., Ltd., personal fees from Meiji Seika Pharma Co., Ltd., personal fees from Pfizer Japan Inc., personal fees from Mochida Pharmaceutical Co., Ltd., grants and personal fees from Shionogi & Co., Ltd., personal fees from Janssen Pharmaceutical K.K., personal fees from Janssen Asia Pacific, personal fees from Yoshitomiyakuhin, personal fees from Astellas Pharma Inc., personal fees from Nippon Boehringer Ingelheim Co. Ltd., personal fees from MSD K.K., personal fees from Kyowa Pharmaceutical Industry Co., Ltd., grants and personal fees from Takeda Pharmaceutical Co., Ltd., personal fees from Taisho Pharmaceutical Co., Ltd., personal fees from Taisho Toyama Pharmaceutical Co., Ltd., grants and personal fees from Eisai Co., Ltd., grants and personal fees from Mitsubishi Tanabe Pharma Corporation, grants from Teijin Pharma, outside the submitted work.

## Supporting information

**Appendix S1** Detailed description of methods.

**Figure S1** Results of enrichment analysis.

**Table S1** Summary of the subject information.

**Table S2** List of neuronal DMRs.

**Table S3** List of nonneuronal DMRs.

**Table S4** Results of gene ontology analysis.
