# Supplementary material for: Cell type‐specific DNA methylation analysis of the prefrontal cortex of patients with schizophrenia
Source: Psychiatry Clin Neurosci. 2021 Jul 22;75(9):297–9. doi: 10.1111/pcn.13282 (PMC8457163; doi:10.1111/pcn.13282)
Supplement: Supplementary file 1 — Appendix S1 Detailed description of methods. [file PCN-75-297-s005.pdf]

Supplementary methods

Postmortem brains

Postmortem brains (prefrontal cortex, Brodmann area 46) from patients with schizophrenia (N = 35) were obtained from the Stanley Medical Research Institute (**Table S1** for demographic variables). The brain set was a part of the Stanley Medical Research Institute Array collection, originally composed of 34 bipolar disorder, 35 schizophrenia, and 35 control brains. Comprehensive DNA methylation analysis of bipolar disorder and control brains was previously carried out (GSE137921)[1], and this study utilized the control dataset for data analysis. This study was approved by the ethics committees of the participating institutes (the Research Ethics Committee of Kumamoto University, the Research Ethics Committee of the Faculty of Medicine of The University of Tokyo, the Ethical Review Board of Juntendo University, and the Wako 1st Research Ethics Committee of RIKEN).

Nuclei isolation

Neuronal and nonneuronal nuclei were separated using NeuN-based nuclei sorting[2, 3]. The nuclear fraction of fresh-frozen brain tissue was retrieved by Percoll discontinuous density gradient centrifugation. Nuclear fractions were then treated with an anti-NeuN antibody (#MAB377, Millipore, Burlington, MA, USA) conjugated with Alexa Fluor 488. NeuN+ and NeuN− nuclei were sorted using a FACS Aria (BD Biosciences, Franklin Lakes, NJ, USA).

Collection of methylated DNA

Methylated DNA was collected by the MBD2B-based method using MethylCollector

(Active Motif, Carlsbad, CA, USA) according to the manufacturer's protocol. A total of 100 ng of extracted genomic DNA was used as the input. Whole-genome amplified human genomic DNA prepared with a GenomiPhi V2 DNA amplification kit (GE Healthcare, Chicago, IL) was used as unmethylated samples. Amplification, probe preparation and labeling were performed according to a previous study[2]. We used Affymetrix human promoter 1.0R tiling arrays (Affymetrix, Santa Clara, CA, USA), which cover 25,500 human promoters by 4.6 million 25-mer oligo probes. Each promoter covers approximately 7.5 kb upstream of the transcription start site to 2.5 kb downstream of the transcription start site with 35 bp probe spacing.

Data analysis

For the purpose of quality control, methylated regions (MRs) were detected by MAT software[4], with each SZ sample as a target and whole-genome amplified unmethylated samples as a reference. After quality control, we omitted three samples from the data analyses. The differentially methylated regions (DMRs) were detected by using deposited data (GSE137921). They were obtained with same MBD2B-based promoter array analysis used for the controls (N = 35). For the controls, duplicate experiments were performed using independently prepared probes (referred to as experiments 1 and 2). In this study, DMRs in schizophrenia were separately identified in experiments 1 and 2. The DMRs detected in experiments 1 and 2 were then intersected by bedtools[5] and used for further analysis. The parameters used in MAT were as follows: bandwidth, 300 bp; max gap, 300; min probe, 10; P-value, 1e-3. Due to differences in the experimental batches between the two studies and the absence of replicated array analysis in this study, we employed a conservative threshold, $P < 1e\text{-}10$, in this study, except we also used P < 1e-

5 for the GWAS overlap test. DMRs on sex chromosomes were excluded from this analysis. Annotation was conducted using HOMER.[6] The DMRs with a MAT score above 1,500 were omitted to avoid possible hybridization artifacts. Gene Ontology (GO) analysis was performed with ToppGene.[7]

We also utilized previously collected gene expression data (Affymetrix GeneChip, HGU133A) obtained from the same brain region from the same sample sets.[8] MAS5-normalized gene expression data were used for analysis according to the manufacturer's instructions (Affymetrix). We identified 59 gene chip probes for 39 DMR-associated genes. Among them, 25 probes for 19 genes were considered to be expressed in the brain based on the flag status provided by MAS5. After adjustment of the brain sample pH,[8] Welch's t-test was conducted for two group comparisons. $P < 0.05$ indicated significance.

To test whether overlap between the DMRs and GWAS loci occurred randomly, we performed a promoter-based random sampling analysis[1]. Based on annotation information from the promoter tiling array (Affymetrix), promoters were assigned as either DMR-overlapping promoters or DMR-nonoverlapping promoters. Then, similarly, promoters were assigned as either GWAS-overlapped promoters or GWAS-nonoverlapped promoters. We considered 102 loci in major depression[9], 108 loci in schizophrenia[10], 30 loci in bipolar disorder[11] and 63 loci in bipolar disorder[12] for this analysis. All loci from each category were compared, and P values were estimated from the probability distribution by 10,000 random samplings of DMR sets using R (v3.5.2).

References

1. Bundo M, Ueda J, Nakachi Y, Kasai K, Kato T, Iwamoto K. Decreased DNA methylation at promoters and gene-specific neuronal hypermethylation in the

prefrontal cortex of patients with bipolar disorder. *Mol Psychiatry* in press. doi: 10.1038/s41380-021-01079-0.

2. Iwamoto K, Bundo M, Ueda J et al. Neurons show distinctive DNA methylation profile and higher interindividual variations compared with non-neurons. *Genome Res* 2011; **21**: 688-96.
3. Bundo M, Kato T, Iwamoto K. Cell Type-Specific DNA Methylation Analysis in Neurons and Glia. **In**: Karpova N (ed.), *Epigenetic Methods in Neuroscience Research*. Springer New York, New York, NY, 2016; 115-123.
4. Johnson WE, Li W, Meyer CA et al. Model-based analysis of tiling-arrays for ChIP-chip. *Proceedings of the National Academy of Sciences of the United States of America* 2006; **103**: 12457-62.
5. Quinlan AR, Hall IM. BEDTools: a flexible suite of utilities for comparing genomic features. *Bioinformatics* 2010; **26**: 841-2.
6. Heinz S, Benner C, Spann N et al. Simple combinations of lineage-determining transcription factors prime cis-regulatory elements required for macrophage and B cell identities. *Mol Cell* 2010; **38**: 576-89.
7. Chen J, Bardes EE, Aronow BJ, Jegga AG. ToppGene Suite for gene list enrichment analysis and candidate gene prioritization. *Nucleic Acids Res* 2009; **37**: W305-11.
8. Iwamoto K, Bundo M, Kato T. Altered expression of mitochondria-related genes in postmortem brains of patients with bipolar disorder or schizophrenia, as revealed by large-scale DNA microarray analysis. *Hum Mol Genet* 2005; **14**: 241-53.
9. Howard DM, Adams MJ, Clarke TK et al. Genome-wide meta-analysis of depression identifies 102 independent variants and highlights the importance of the prefrontal brain regions. *Nature neuroscience* 2019; **22**: 343-352.
10. Schizophrenia Working Group of the Psychiatric Genomics C. Biological insights from 108 schizophrenia-associated genetic loci. *Nature* 2014; **511**: 421-7.
11. Stahl EA, Breen G, Forstner AJ et al. Genome-wide association study identifies 30 loci associated with bipolar disorder. *Nature genetics* 2019; **51**: 793-803.
12. Mullins N, Forstner AJ, O'Connell KS et al. Genome-wide association study of over 40,000 bipolar disorder cases provides novel biological insights. *medRxiv* 2020.
