# Supplementary material for: Cell type‐specific DNA methylation analysis of the prefrontal cortex of patients with schizophrenia
Source: Psychiatry Clin Neurosci. 2021 Jul 22;75(9):297–9. doi: 10.1111/pcn.13282 (PMC8457163; doi:10.1111/pcn.13282)
Supplement: Supplementary file 2 — Figure S1 Results of enrichment analysis. [file PCN-75-297-s004.pdf]

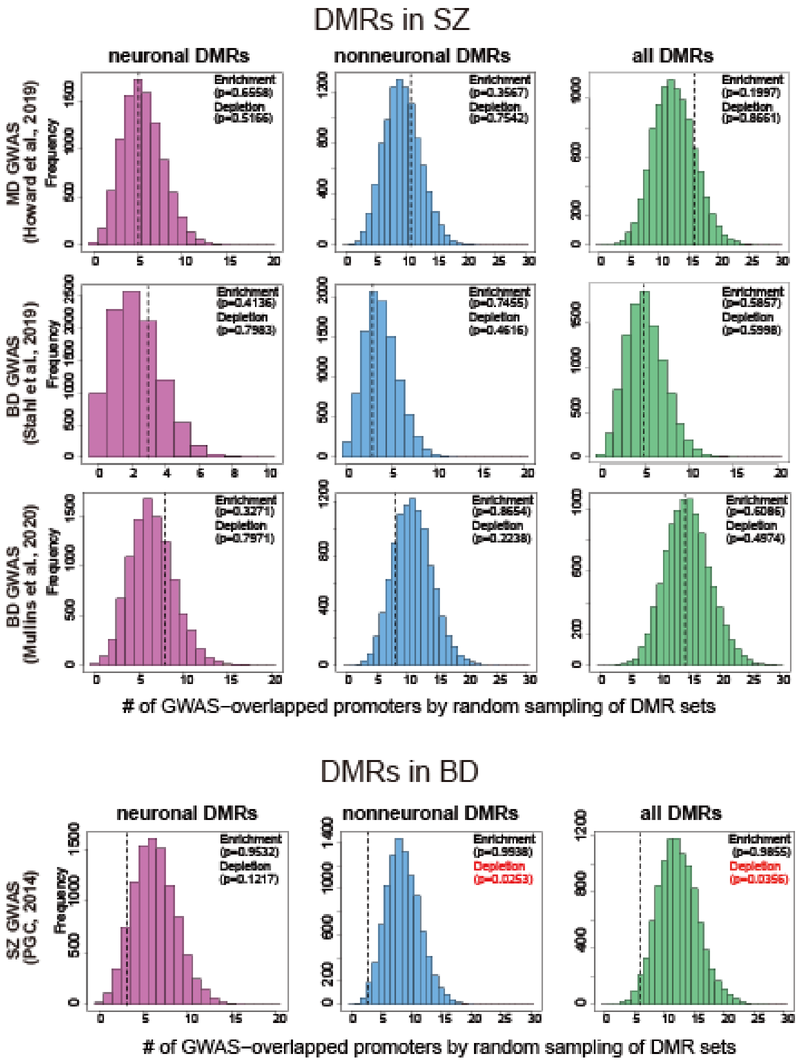

Figure S1. Enrichment and depletion test between the DMRs and the GWAS loci by random sampling. DMRs in SZ were identified in this study, while those in BD were previously identified (Bundo et al., 2021). Frequency was based on 10,000 random sampling of DMR sets. P value given in red indicates significant depletion of DMRs. Note that the results of DMRs in BD were based on the independent random sampling, and were consistent with a previous study (Bundo et al., 2021). DMRs, differentially methylated regions; GWAS, genome-wide association study; SZ, schizophrenia; MD, major depression, BD, bipolar disorder.
